# Prevalence of non-communicable diseases, multimorbidity, and their impact on activity limitations among adults with chronic back pain: a national population-based study in a middle-income country

**DOI:** 10.1016/j.bjpt.2025.101241

**Published:** 2025-08-05

**Authors:** Érica de Matos Reis Ferreira, Ítalo Ribeiro Lemes, Edmar Geraldo Ribeiro, Deborah Carvalho Malta, Christopher G. Maher, James H. McAuley, Matthew D. Jones, Luiz Hespanhol, Rafael Zambelli Pinto

**Affiliations:** a Department of Physical Therapy, Universidade Federal de Minas Gerais (UFMG), Belo Horizonte, MG, Brazil; b Department of Physical Therapy, Universidade Estadual Paulista (UNESP), Presidente Prudente, Brazil; c Department of Maternal Child Nursing and Public Health, School of Nursing, Universidade Federal de Minas Gerais (UFMG), Belo Horizonte, Brazil; d Institute for Musculoskeletal Health, Sydney Local Health District, Sydney, Australia; e Sydney School of Public Health, Faculty of Medicine and Health, University of Sydney, Sydney, Australia; f Centre for Pain IMPACT, Neuroscience Research Australia, Sydney, New South Wales, Australia; g School of Health Sciences, Faculty of Medicine and Health, University of NSW, Sydney, New South Wales, Australia; h Department of Physical Therapy, Faculty of Medicine, University of Sao Paulo (USP), Sao Paulo, Brazil; i Discipline of Physiotherapy, Graduate School of Health, Faculty of Health, University of Technology, Sydney, New South Wales, Australia

**Keywords:** Back pain, Comorbidities, Disability, Multimorbidity, Non-communicable diseases

## Abstract

**Background:**

Patients with chronic back pain who also have comorbidities are more likely to report severe symptoms and receive inappropriate care. It is still unclear which types of comorbidity are associated with more disabling symptoms.

**Objective:**

To compare the prevalence of chronic non-communicable disease between adults with and without chronic back pain, estimate the prevalence of multimorbidity in adults with chronic back pain, and identify comorbidities that are associated with activity limitations.

**Methods:**

Data from adults who participated in the 2019 National Health Survey were used to collect information about chronic non-communicable diseases, chronic back pain, and activity limitations. Multimorbidity was defined as the presence of one or more comorbidities in addition to chronic back pain. Difference in proportion and ordinal logistic regression were used in the analyses.

**Results:**

Of the total sample size (*n* = 87,678), 21.6 % (*n* = 18,930) reported having chronic back pain. The prevalence rates of all non-communicable conditions were higher in people with chronic back pain compared to those without chronic back pain. Multimorbidity was reported by 62.1 % (95 %CI: 61.1, 63.6) of the adults with chronic back pain. The odds of a participant with comorbidities reporting a worse activity limitation level were 2.1 (95 %CI:1.9, 2.4) for arthritis or rheumatism, 1.6 (95 %CI: 1.4, 1.8) for depression, and 1.5 (95 %CI: 1.3, 1.6) for cardiovascular disease compared to participants without this specific comorbidity.

**Conclusion:**

Non-communicable diseases and multimorbidity were prevalent in adults with chronic back pain. Cardiovascular disease, arthritis or rheumatism, and depression, were associated with higher activity limitations due to back pain.

## Highlights

• Six in every ten people with chronic back pain have one or more comorbidities.
• Comorbidities are more prevalent in people with chronic back pain than in those without chronic back pain.
• Cardiovascular disease, arthritis and depression are associated with activity limitations.

## Introduction

Multimorbidity, or the coexistence of multiple health conditions in the same individual, imposes a challenge to healthcare clinicians.^1^ Nine out of 10 patients seen in primary care settings have more than one health condition.^2^^,^^3^ Compared to those with a single condition, those with multimorbidity have lower quality of life^4^ and poorer function,^5^ are more likely to die prematurely,^6^ and consume more healthcare resources.^7^ Disease management of patients with multimorbidity and chronic pain can become more complex for pharmacological interventions, as dosing and adverse effect profiles of pain medication can be affected by other diseases,^8^ and non-pharmacological interventions, such as exercise therapy, as modality and intensity recommended for a specific disease may be contra-indicated or not suitable for other diseases.

Chronic back pain is ranked as the leading cause of disability worldwide.^9^ Although clinical practice guidelines advise the condition should be managed in primary care,^10^ most available evidence used in guidelines comes from trials that did not consider, or excluded people with associated health conditions.^10^ There is evidence, mainly from upper income countries, suggesting that chronic non-communicable conditions frequently coexist with chronic back pain,^11^ such as obesity,^12^ depression,^13^ hypertension,^14^, ^15^, ^16^ diabetes,^17^ chronic obstructive pulmonary disease,^18^ asthma,^11^ arthritis^19^ and cardiovascular disease.^11^^,^^20^^,^^21^ Patients with back pain and multimorbidity are more likely to have worse symptoms,^22^^,^^23^ show poorer prognosis,^24^ and receive inappropriate care.^25^

Previous research in the back pain field focused on the number of comorbidities. Cohort studies have shown that comorbidities are associated with poor prognosis in patients with back pain.^22^^,^^26^^,^^27^ While some population-based studies have provided information on comorbidities commonly associated with chronic back pain, these studies were predominantly conducted in upper-income countries^16^^,^^28^^,^^29^ where rates and impact of comorbidity may differ from middle- or lower-income countries (LMICs).^30^ Population-based studies conducted so far in LMICs focused broadly on multimorbidity (i.e., not specifically related to back pain),^31^^,^^32^ or a specific comorbidity (i.e., mental health problems) associated with back pain.^33^, ^34^, ^35^ Multimorbidity imposes a financial burden on LMICs^36^ and is complicated by multiple exacerbating factors including adverse environmental and early life stressors linked to poverty, limited social infrastructure and poorer family coping mechanisms that translate into chronic diseases occurring at earlier ages.^37^ Knowledge about types of comorbidities associated with more disabling back symptoms would enable researchers and clinicians to develop and test management strategies tailored to various comorbidity profiles.

Brazil, a middle-income country, is facing a significant challenge with both back pain^38^ and multimorbidity.^21^ The 2019 National Health Survey was a population-based study which recruited a representative sample of the Brazilian population and can yield generalisable estimates on comorbidities. Therefore, we aimed to:

i) compare the prevalence of non-communicable disease in people with and without chronic back pain;
ii) estimate the prevalence of multimorbidity among people with chronic back pain;
iii) characterise and contrast the sociodemographic profile among people with back pain who reported having only chronic back pain, multimorbidity and each comorbidity associated with chronic back pain, and
iv) identify comorbidities associated with activity limitation due to chronic back pain.

## Methods

### Study design and participants

This is a secondary analysis of data from the 2019 National Health Survey conducted by the Brazilian Institute of Geography and Statistics (abbreviated as IBGE) in partnership with the Brazilian Ministry of Health. The 2019 National Health Survey is a household survey that employs a complex sampling design, ensuring representation of the Brazilian population residing in private households in urban and rural areas across the five geographic macro-regions of Brazil. The census tracts or sets of sectors formed the primary sampling units, and households constituted the second-stage units, and residents aged 15 years or older were defined as the units of the third stage.^39^^,^^40^ More details on the study methods are available elsewhere.^39^ Data collection for the 2019 National Health Survey occurred between August 26, 2019, and March 13, 2020. Hand-held computers were used to collect survey information and all interviewers received training. The interviewer explained to residents the objectives, the data collection procedures and the importance of their participation in the research. The National Health Survey results are published in IBGE through descriptive analyses of main health indicators in mass media circulation so that the population is aware of the main results. A total of 88,531 adult respondents participated in the 2019 National Health Survey. The data used for the current study were obtained through microdata publicly accessible on the National Health Survey (NHS) website.^41^

The present study used data from all adult individuals 18 years of age or older, who responded to the Chronic Diseases Module of the 2019 National Health Survey. The National Health Survey defines chronic in the survey questionnaire pack as conditions that last >6 months. All adults who provided self-reported information about their weight and height were included so that body mass index (BMI) could be calculated. Additionally, all participants received an explanation on the concept of chronic disease and signed an informed consent form before data collection.

### Variables

#### Sociodemographic and lifestyle

The sociodemographic and lifestyle variables in the data set included: sex (according to biological characteristics),^42^ age groups, education levels, per capita household income, location of residence, presence of private health plan, smoking, abusive alcohol consumption; BMI, calculated by the weight and height reported by the respondent.

#### Presence and duration of chronic back pain

The presence of chronic back pain was assessed by the following question: “*Do you have chronic back problems, such as chronic pain in your back or neck, lumbago, sciatica, or problems in the vertebrae or discs?*” The response options were “*yes*” or “*no*”. For affirmative responses regarding the presence of chronic back pain, respondents answered a follow-up question about the age when the current back pain started. Symptom duration was calculated by subtracting the respondent’s age when back pain started from the age at the time of the survey. Symptom duration was categorised as greater than 1 year and equal or less than a year.

#### Presence of chronic non-communicable conditions

The presence of non-communicable disease was identified through the question: “*Has any doctor ever diagnosed you with [disease]?*” The chronic non-communicable conditions investigated included: hypertension, heart disease, cerebrovascular accident (stroke), diabetes, asthma, arthritis or rheumatism, cancer, depression, other mental health conditions and other lung diseases. The responses were dichotomous (yes or no). Participants with and without chronic back pain who responded affirmatively to at least one chronic condition were categorised as having a chronic non-communicable condition (or comorbidity). For analysis purposes, hypertension, heart disease, and stroke were categorised into cardiovascular diseases.^20^^,^^43^ The presence of multimorbidity^1^^,^^44^ among participants with chronic back pain was determined based on the positive response to one or more comorbidities in addition to the presence of chronic back pain (chronic back pain + 1 or more chronic conditions) which could range from two to five comorbidities. Females who reported the presence of diabetes and/or hypertension only during pregnancy were not included as hypertensive and/or diabetic individuals. Individuals who declared that they had never had their blood pressure checked or their blood glucose tested were not considered to have hypertension or diabetes, respectively.

#### Outcome variables

Activity limitation due to chronic back pain was assessed with the question: “*In general, to what extent does your back problem limit your usual activities (such as working, household chores, etc.?)*”. The response options included: (1) no limitation; (2) mild limitation; (3) moderate limitation; (4) severe limitation; (5) very severe limitation.^15^ Supplementary material – Table 1 describes the variables investigated in this study.

**Table 1:** Description of study variables in the population with and without chronic back pain.

Characteristics	Chronic back pain	
	*No* (*n* = 68,748)	*Yes* (*n* = 18,930)
**Sex**		
Female	50.7 (49.9, 51.3)	60.0 (58.8, 61.2)
**Age groups (years)**		
Mean (95 % CI)	43.3 (43.1, 43.6)	51.3 (50.8, 51.7)
18–29	24.9 (24.2, 25.6)	10.2 (9.3, 11.2)
30–39	22.4 (21.8, 23.0)	15.1 (14.2, 16.0)
40–49	17.7 (17.2, 18.3)	20.0 (18.9, 21.1)
50–59	15.6 (15.1, 16.0)	23.2 (22.2, 24.3)
60–69	10.8 (10.4, 11.2)	17.6 (16.6, 18.4)
70–79	5.7 (5.4, 5.9)	9.7 (9.0, 10.4)
80 and more	2.6 (2.4, 2.8)	3.9 (3.5, 4.4)
**Educational levels**		
Illiterate or incomplete primary school	31.5 (30.8, 32.2)	47.0 (45.7, 48.4)
Complete primary school or incomplete high school	15.0 (14.5, 15.5)	12.0 (11.2, 12.9)
Complete secondary school or tertiary education incomplete	36.9 (36.2,37.6)	27.1 (25.9, 28.4)
Tertiary education complete	16.4 (15.7,17.1)	13.6 (12.6, 14.7)
**Household income**		
≤1 minimum wage	50.8 (49.9, 51.6)	52.0 (50.5, 53.4)
> 1 and ≤ 3 minimum wage	37.4 (36.7, 38.1)	37.0 (35.7, 38.4)
> 3 and ≤ 5 minimum wage	6.5 (6.1, 6.9)	5.6 (5.0, 6.1)
> 5 minimum wage	5.1 (4.7, 5.5)	5.2 (4.6, 5.8)
**Household situation**		
Urban	86.5 (86.1, 86.9)	84.7 (83.8, 85.6)
**Health Insurance**		
Yes	26.8 (26.0, 27.6)	27.8 (26.4, 29.3)
**Smoking**		
Non-smoking	62.9 (62.3, 63.6)	52.0 (50.6, 53.4)
Ex-smoker	24.7 (24.2, 25.3)	33.8 (32.5, 35.1)
Smoker	12.2 (11.7, 12.6)	14.0 (13.2, 14.9)
**Abusive alcohol consumption**		
Yes	2.3 (2.1, 2.5)	3.1 (2.6, 3.6)
**BMI (kg/m^2^)**		
Mean (95 % CI)	26.3 (26.3, 26.4)	27.1 (27.0, 27.3)
**Duration of chronic back pain**		
≤1 year	–	3.9 (3.4, 4.4)
>1 year	–	96.0 (95.5, 96.5)
**Activity limitation due to chronic back pain**		
No limitation	–	31.8 (30.4, 33.1)
Mild limitation	–	32.8 (31.6, 34.1)
Moderate limitation	–	19.1 (18.1, 20.2)
Severe limitation	–	11.8 (10.9, 12.8)
Very severe limitation	–	4.2 (3.7, 4.7)

Data are proportions (95 % confidence interval), unless otherwise indicated. BMI, body mass index.

Sample size include 68,748 people without chronic back pain (population size [N] = 157,559,876) and 18,930 with back pain (population size [N] = 34,131,360). Proportions or means with 95 % Confidence Interval (CI) incorporate appropriate weights to control complex sample design.

#### Ethical aspects

2019 National Health Survey data are available for public access and use; the current study was approved by the Comissão Nacional de Ética e Pesquisa (CONEP) Distrito Federal, Brasília, Brazil (n° 3529,376, 2019).

#### Data analysis

To characterise the sample, we presented sociodemographic characteristics, lifestyle, chronic conditions, and the presence of multimorbidity using proportions and 95 % confidence intervals (CI), means with 95 %CI, or median with interquartile range (IQR), depending on the distribution of the data. Multimorbidity clusters were depicted as proportions and 95 %CI for the total sample and stratified by sociodemographic characteristics and activity limitation due to chronic back pain. Sociodemographic data were chosen for comparison (i.e., sex, age, education levels, smoking, alcohol consumption) because these are more prevalent in specific groups^14^^,^^45^^,^^46^ and are known lifestyle risk factors for back pain^45^^,^^46^ or other chronic conditions.^47^ Comparisons within and between comorbid groups were conducted using mean difference (MD) and proportion difference (PD), along with their respective 95 %CI.

We employed an ordinal logistic regression model^48^ to identify which type of comorbidity was associated with activity limitation due to chronic back pain. The outcome variable was the level of activity limitation due to chronic back pain with “no limitation” serving as the reference category. We estimated the odds of moving to a higher category of activity limitation due to chronic back pain for each identified comorbidity group. We presented the adjusted odds ratio for the association of each comorbid group with activity limitation controlled for the presence of all comorbidities. We did not control for other confounding variables because making causal inferences was beyond the scope of this study. The 95 %CI was reported for each estimate. For comparison purposes only, we estimated the odds of moving to a higher category of activity limitation due to chronic back pain for the no-comorbidities back pain group. Data were organised and analysed using Stata® software, version 15.1, through the survey module, which considers the effects of complex sampling.^39^^,^^41^^,^^49^ Expansion factors and sampling weights were considered in all analyses. Data from the National Health Survey are collected with a complex sampling design; therefore, statistical analysis was carried out with an application that considers the effect of the sampling plan and unequal selection probabilities. Because of weighting, it is expected that there will be differences between the prevalence estimates calculated using the study sample and the prevalence estimates calculated with weighting.

## Results

### Participants characteristics

Among the total participants (*n* = 87,678), 21.6 % (*n* = 18,930) reported chronic back pain. Participants with chronic back pain were older (MD: 6.7 years, 95 %CI: 6.5, 7.0), presented with higher BMI (MD: 0.5 kg/m^2^, 95 %CI:0.4, 0.6) and showed higher proportions of females (PD: 10.0 %, 95 %CI: 8.9, 11.0) and of participants in the illiterate or incomplete primary school (PD: 15.5 %, 95 %CI: 14.3, 16.6) than those participants without chronic back pain. Specifically for the chronic back pain group, most participants (96.0 %, 95 %CI: 95.5, 96.5) reported experiencing symptoms for more than one year. Two-thirds of the people with chronic back pain presented with some activity limitations (68.2 %, 95 %CI: 66.8, 69.5) (i.e., scored at equal to or greater than mild activity limitation). Of these, 32.8 % (95 %CI: 31.6, 34.1) of participants reported having mild activity limitation and 35.2 % (95 %CI: 33.8, 36.7) reported “moderate” to “very severe” limitation (Table 1).

### Prevalence of chronic non-communicable conditions and multimorbidity

**Fig. 1:**
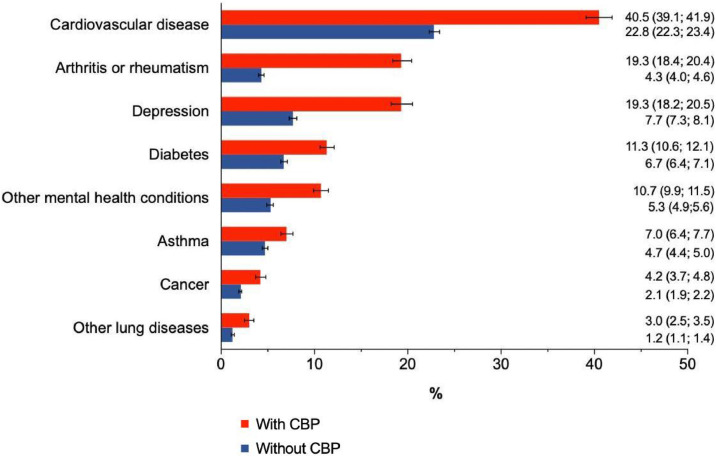
Prevalence of comorbidities investigated in the population with and without chronic back pain, National Health Survey, 2019. CBP, chronic back pain.

The prevalence rates of chronic non-communicable conditions are described in Fig. 1. The top three most prevalent non-communicable diseases in people without chronic back pain were cardiovascular diseases (22.8 %; *n* = 17,179), followed by depression (7.7 %; *n* = 5001) and diabetes (6.7 %; *n* = 5038). The order of prevalence changed in those people with chronic back pain with cardiovascular diseases (40.5 %; *n* = 7666), arthritis or rheumatism (19.3 %; *n* = 3678), and depression (19.3 %; *n* = 3179) being the top three most prevalent conditions. The prevalence rates of all non-communicable conditions were higher in people with chronic back pain, particularly for cardiovascular disease (PD:17.7 %, 95 %CI:16.4, 18.9), arthritis or rheumatism (PD:15.0 %, 95 %CI: 13.5, 16.4) and depression (PD: 11.6 %, 95 %CI: 10.0, 13.1), compared to those without chronic back pain. The prevalence rate of multimorbidity in those with chronic back pain was 62.1 % (95 %CI: 61.1, 63.6; *n* = 11,608).

### Sociodemographic profile among people with chronic back pain

Among those with chronic back pain, we found that the no-comorbidities back pain group and the multimorbidity group were relatively similar regarding smoking, and abusive alcohol consumption; but not for sex, age, education level, and BMI (Table 2). Regarding sex, a higher proportion of females was found in the multimorbidity group compared to the no-comorbidities back pain group (PD=20.8 %, 95 %CI: 18.6, 22.8). The proportions of females were higher across all comorbidity groups. For instance, in comparison with the no-comorbidities chronic back pain group, the group reporting comorbid depression showed the highest proportion of females (PD=34.2 %, 95 %CI: 31.9, 36.4), and the group reporting other lung diseases as comorbidity showed the smallest proportion of females (PD=20.3 %, 95 %CI: 15.0, 25.7).

**Table 2:** Sociodemographic characteristics for adults with chronic back pain according to presence and types of comorbidities.

Characteristics	No-comorbidities chronic back pain group (*n* = 7322)	Multimorbidity (*n* = 11,608)	Comorbidities							
			Cardiovascular disease (*n* = 7666)	Arthritis or rheumatism (*n* = 3678)	Depression (*n* = 3179)	Diabetes (*n* = 2038)	Other mental health conditions (*n* = 1739)	Asthma (*n* = 1298)	Cancer (*n* = 759)	Other lung disease (*n* = 525)
**Sex,***Female*	46.9 (44.8, 49.1)	67.8 (66.4, 69.2)	66.3 (64.6, 68.1)	79.3 (76.9, 81.5)	81.1 (78.7, 83.4)	68.1 (64.3, 71.7)	78.2 (74.9, 81.2)	71.8 (67.1, 76.1)	69.8 (64.0, 75.1)	64.3 (57.5, 70.6)
**Age***, years*										
Mean (95 %CI)	43.5 (42.9, 44.1)	55.9 (55.3, 56.5)	59.8 (59.1, 60.6)	60.4 (59.6, 61.2)	52.1 (51.1, 53.2)	61.5 (60.5, 62.6)	47.6 (46.3, 48.8)	49.3 (47.7, 50.9)	62.0 (60.4, 63.6)	56.7 (54.3, 59.0)
**18–29**	18.5 (16.6, 20.5)	5.3 (4.5, 6.1)	2.0 (1.5, 2.7)	1.2 (0.7, 2.1)	6.5 (5.0, 8.4)	0.5 (0.2, 1.2)	12.5 (9.8, 16.0)	13.8 (10.3, 18.2)	0.4 (0.0, 2.2)	7.6 (4.9, 11.7)
**30- 39**	24.1 (22.4, 25.9)	9.6 (8.7, 10.7)	6.2 (5.19, 7.58)	5.5 (4.2, 7.2)	12.8 (10.7, 15.2)	5.0 (3.3, 7.5)	17.9 (15.1, 21.0)	15.7 (12.6, 19.5)	4.8 (2.7, 8.3)	6.0 (3.6, 9.8)
**40–49**	23.5 (21.9, 25.3)	17.8 (16.4, 19.4)	14.3 (12.5, 16.5)	12.5 (10.7, 14.6)	22.8 (20.1, 25.7)	11.1 (7.9, 15.3)	24.3 (21.2, 27.8)	20.4 (15.8, 26.0)	13.3 (9.3, 18.7)	17.8 (10.0, 29.7)
**50–59**	20.5 (18.8, 22.2)	24.9 (23.5, 26.3)	25.2 (23.4, 27.0)	27.4 (25.0, 30.0)	26.9 (24.0, 30.0)	24.6 (21.5, 28.1)	23.8 (20.8, 27.1)	20.6 (17.0, 24.7)	21.3 (17.0, 26.8)	24.0 (17.6, 31.9)
**60–69**	8.5 (7.6, 9.5)	22.9 (21.6, 24.2)	26.4 (24.6, 28.2)	28.4 (26.0, 30.9)	19.5 (17.2, 22.2)	31.8 (28.2, 35.6)	14.5 (12.1, 17.3)	17.9 (14.8, 21.4)	29.2 (23.6, 35.6)	20.5 (15.1, 27.2)
**70–79**	3.5 (2.9, 4.2)	13.5 (12.5, 14.6)	17.8 (16.4, 19.4)	17.3 (15.4, 19.3)	8.5 (7.0, 10.2)	20.2 (17.6, 22.9)	5.4 (3.9, 7.3)	7.3 (5.7, 9.3)	22.9 (18.1, 28.6)	16.5 (11.6, 22.9)
**≥80**	1.1 (0.8, 1.5)	5.6 (5.0, 6.4)	7.7 (6.7, 8.8)	7.3 (6.0, 8.9)	2.7 (2.0, 3.7)	6.5 (4.8, 8.6)	1.2 (0.7, 2.0)	3.9 (2.6, 5.7)	7.7 (5.4, 10.9)	7.2 (4.7, 10.9)
**Education levels**										
**Illiterate or incomplete primary school**	39.1 (37.1, 41.2)	51.8 (50.1, 53.5)	59.0 (56.8, 61.1)	57.0 (54.3, 59.7)	44.6 (41.7, 47.6)	64.6 (60.8, 68.3)	32.9 (29.3, 36.7)	44.3 (39.2, 49.5)	46.0 (40.0, 52.2)	54.9 (46.4, 63.1)
**Complete primary school or incomplete high school**	13.7 (12.3, 15.2)	11.0 (10.0, 12.0)	10.8 (9.6, 12.1)	11.5 (9.7, 23.5)	12.5 (10.3, 14.9)	9.6 (7.5, 12.1)	14.2 (11.5, 17.3)	12.9 (10.0, 16.5)	12.8 (8.4, 19.0)	13.4 (9.1, 19.2)
**Complete secondary school or tertiary education incomplete**	32.5 (30.4, 34.6)	23.9 (22.4, 25.5)	19.9 (18.0, 21.9)	20.7 (18.5, 23.2)	25.8 (23.4, 28.5)	18.5 (15.5, 21.9)	32.8 (29.4, 36.4)	27.5 (23.4, 32.1)	25.1 (20.1, 30.6)	24.7 (18.0, 32.9)
**Tertiary education complete**	14.5 (12.9, 16.2)	13.1 (11.9, 14.4)	10.1 (8.8, 11.6)	10.6 (9.0, 12.4)	16.9 (14.6, 19.4)	7.1 (5.5, 9.1)	19.9 (17.2, 23.1)	15.1 (12.3, 18.4)	15.9 (12.2, 20.5)	6.8 (4.8, 9.7)
**BMI, *kg/m^2^*** Mean (95 %CI)	26.1 (25.9, 26.3)	27.8 (27.5, 28.0)	28.3 (28.0, 28.6)	28.0 (27.7, 28.4)	28.1 (27.6, 28.7)	29.4 (28.8, 30.0)	27.7 (27.2, 28.2)	27.9 (26.9, 29.0)	27.8 (27.1, 28.6)	27.7 (25.7, 29.7)
**Smoking**										
**Non smoker**	58.1 (55.9, 60.2)	48.4 (46.6, 50.3)	47.0 (44.6, 49.4)	48.9 (46.1, 51.6)	51.9 (48.3, 55.4)	46.2 (42.3, 50.2)	49.5 (45.5, 53.4)	47.4 (42.2, 52.2)	45.4 (39.2, 51.7)	34.8 (25.9, 44.9)
**Former smoker**	26.0 (24.2, 27.9)	38.5 (36.8, 40.2)	41.0 (38.8, 43.3)	39.0 (36.2, 41.7)	34.0 (30.8, 37.3)	44.1 (40.2, 48.1)	35.5 (31.8, 39.4)	36.0 (31.3, 40.9)	39.2 (33.6, 45.1)	45.5 (36.8, 54.4)
**Smoker**	15.8 (14.3, 17.4)	12.9 (11.9, 14.0)	11.8 (10.7, 13.1)	12.0 (10.3, 14.0)	14.0 (12.0, 16.2)	9.5 (7.4, 12.0)	14.8 (12.4, 17.7)	16.5 (13.1, 20.6)	15.3 (10.9, 20.9)	19.6 (14.1, 26.4)
**Abusive consumption of alcohol**										
**Yes**	3.5 (2.8, 4.4)	2.8 (2.3, 3.5)	2.8 (2.2, 3.6)	2.2 (1.4, 3.4)	1.8 (1.2, 2.7)	2.1 (1.3, 3.3)	1.9 (1.1, 3.0)	4.0 (2.0, 7.8)	3.3 (1.2, 8.6)	2.9 (1.4, 5.9)
**Activity limitation due to chronic back pain**										
**No limitation**	41.5 (39.2, 43.7)	25.9 (24.4, 27.4)	24.1 (22.3, 25.9)	15.8 (13.8, 18.2)	22.5 (19.8, 25.4)	22.5 (19.3, 26.0)	27.6 (23.9, 31.6)	28.2 (24.2, 32.6)	29.6 (23.9, 35.9)	20.1 (14.8, 26.7)
**Mild limitation**	32.0 (30.0, 34.1)	33.3 (31.7, 35.0)	33.4 (31.4, 35.5)	31.2 (28.6, 34.0)	29.1 (26.2, 32.3)	34.3 (30.6, 38.1)	29.4 (26.0, 33.1)	28.0 (24.2, 32.3)	30.6 (25.2, 36.5)	26.7 (20.3, 34.3)
**Moderate limitation**	17.2 (15.4, 19.3)	20.3 (19.0, 21.6)	21.0 (19.4, 22.7)	25.6 (23.2, 28.0)	23.3 (20.5, 26.3)	19.3 (16.5, 22.3)	21.3 (18.3, 24.7)	20.0 (16.3, 24.3)	17.0 (13.3, 21.5)	18.5 (13.6, 24.8)
**Severe limitation**	7.1 (6.2, 8.1)	14.7 (13.4, 16.1)	15.5 (13.8, 17.4)	19.1 (17.2, 21.2)	17.6 (15.6, 19.9)	17.0 (13.8, 20.8)	15.0 (12.5, 17.8)	17.0 (12.7, 22.4)	16.2 (12.0, 21.5)	22.8 (14.8, 33.4)
**Very severe limitation**	1.9 (1.5, 2.5)	5.6 (4.9, 6.4)	5.7 (4.9, 6.7)	8.0 (6.6, 9.6)	7.3 (5.7, 9.2)	6.7 (5.0, 9.0)	6.4 (4.8, 8.6)	6.4 (4.4, 9.3)	6.4 (3.8, 10.6)	11.5 (7.2, 18.0)

Data are proportion (95 % confidence interval) unless otherwise indicated. BMI, body mass index.

Sample size without CBP (n) 68,748 and with CBP (n) 18,930; Population size (N) 34,131,360.

Proportions or means with 95 % Confidence Interval (CI) incorporate appropriate weights to control complex sample design.

The mean age was 12.1 (95 %CI: 11.7, 12.5) years higher in the multimorbidity group compared to the no-comorbidities chronic back pain group. Among the comorbidity groups, the largest age differences were found for the group reporting comorbid cancer (MD=18.2 years, 95 %CI: 17.1, 19.2) and for the group reporting comorbid diabetes (MD=17.5 years, 95 %CI: 16.8, 18.2) compared to the no-comorbidities chronic back pain group. Regarding education level, a higher proportion of illiterate participants or participants with incomplete primary school was found in the multimorbidity group compared to the no-comorbidities chronic back pain group (PD: 9.2, 95 %CI: 7.2, 11.3). This finding was similar across all comorbidity groups.

The mean BMI was 1.4 kg/m^2^ (95 %CI:1.3, 1.6) higher in the multimorbidity group compared to the no-comorbidities chronic back pain group. Among the comorbidity groups, the largest BMI differences were found for the group reporting comorbid diabetes (MD=2.6 kg/m^2^, 95 %CI:2.4, 2.9) and for the group reporting comorbid cardiovascular disease (MD=1.9 kg/m^2^, 95 %CI:1.8, 2.1) compared to the no-comorbidities chronic back pain group.

### Level of activity limitation associated with multimorbidity/comorbidities

**Fig. 2:**
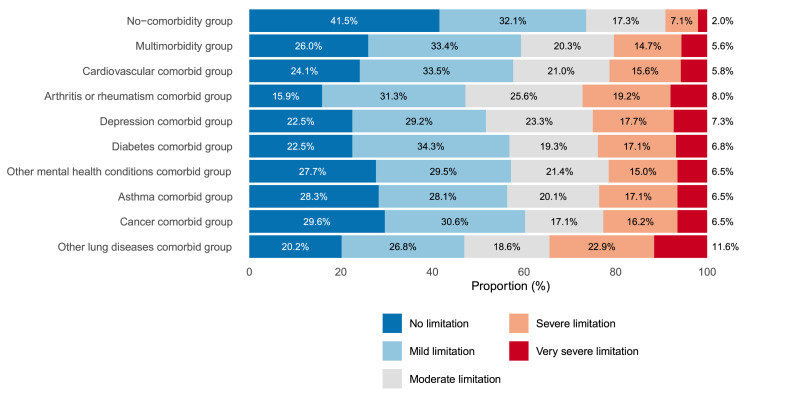
Level of activity limitation by comorbidity among population with chronic back pain, National Health Survey, 2019.

Fig. 2 presents the proportion of people in each activity limitation category across all groups. Differences in proportions between the no-comorbidities chronic back pain group and the multimorbidity group were more evident in the no activity limitation category and the severe and very severe activity limitation categories. While the no-comorbidities chronic back pain group showed a higher proportion of participants reporting no activity limitation (PD=15.5 %, 95 %CI:13.1, 18.0) compared to the multimorbidity, the multimorbidity group showed higher proportions of participants reporting severe (PD=7.5 %, 95 %CI: 4.9, 10.2) and very severe (PD=3.6 %, 95 %CI: 0.8, 6.3) activity limitation. All comorbidity groups showed similarly low proportions of participants with no activity limitation and higher proportions at higher activity limitation levels, particularly for severe and very severe activity limitation categories (Table 2).

The results of the ordinal logistic regression models indicate that the odds of a participant with a specific comorbidity reporting a worse activity limitation level were 2.1 (95 %CI: 1.9, 2.4) for arthritis or rheumatism, 1.8 (95 %CI: 1.3, 2.5) for other lung disease, 1.6 (95 %CI: 1.4, 1.8) for depression, 1.5 (95 %CI: 1.3, 1.6) for cardiovascular disease, and 1.3 (95 %CI: 1.1, 1.5) for diabetes compared to a participant without this specific comorbidity. No association with activity limitation was found for the comorbidities of asthma 1.1 (95 %CI: 0.9, 1.3), cancer 1.0 (95 %CI: 0.7, 1.3) and other mental health conditions 1.0 (95 %CI: 0.9, 1.2) (Table 3).

**Table 3:** Association between activity limitation and comorbidity groups among people with chronic back pain.

Chronic conditions	OR (95 % CI)
Chronic back pain with no-comorbidities ^a^	
*No*	1.0
*Yes*	0.4* (0.4, 0.5)
Arthritis or rheumatism	
*No*	1.0
*Yes*	2.1* (1.9, 2.4)
Other lung disease	
*No*	1.0
*Yes*	1.8* (1.3, 2.5)
Depression	
*No*	1.0
*Yes*	1.6* (1.4, 1.8)
Cardiovascular disease	
*No*	1.0
*Yes*	1.5* (1.3, 1.6)
Diabetes	
*No*	1.0
*Yes*	1.3* (1.1, 1.5)
Asthma	
*No*	1.0
*Yes*	1.1 (0.9, 1.3)
*Cancer*	
*No*	1.0
*Yes*	1.0 (0.7, 1.3)
Other mental health conditions	
*No*	1.0
*Yes*	1.0 (0.9, 1.2)

Note: OR, odds ratio; 95 % CI, 95 % confidence interval; CBP, chronic back pain.

Activity limitation due to chronic back pain (0: no limitation; 1: mild; 2: moderate; 3: severe; 4: very severe). *^a^ was included only for comparison and was not included in the multiple ordinal analysis*.

Sample size (n) 18,930. Population size (N) 34,131,360.

⁎ Indicate significant associations (*p* < 0.05).

## Discussion

This study using national population-based data revealed that non-communicable diseases are more prevalent in adults with chronic back pain compared to adults without chronic back pain. While the most prevalent conditions among adults with chronic back pain were cardiovascular disease (40.5 %, 95 %CI: 39.1, 41.9), arthritis or rheumatism (19.3 %, 95 %CI: 18.2, 20.5) and depression (19.3 %, 95 %CI: 18.4, 20.4), the conditions more prevalent in adults without chronic back pain were cardiovascular disease (22.8 %, 95 %CI: 22.3, 23.4), depression (7.7 %, 95 %CI: 7.3, 8.1), and diabetes (6.7 %, 95 %CI: 6.4, 7.1). Our findings also showed that comorbidity is very common among Brazilian adults with chronic back pain. Six in every 10 people with chronic back pain also report having at least one other chronic health condition. These people are more likely to be females, older, obese, have low education levels and report more activity limitation than those with chronic back pain but no comorbidities. The comorbidities most likely to be associated with activity limitation in people with chronic back pain were arthritis or rheumatism, followed by other lung disease, depression, cardiovascular disease and diabetes.

Our findings confirmed previous evidence from upper income countries that adults with chronic back pain have a higher prevalence of chronic non-communicable conditions than those without back pain. Comparison between our findings and these previous studies should be done with caution due to differences in symptoms duration and chronic conditions investigated. Data from Germany shows a higher prevalence of osteoarthritis (50 % versus 21 %), hypertension (26 % versus 21 %), myocardial infarction (3 % versus 2 %), cancer (5 % versus 3 %) and diabetes (3.5 % versus 2.5 %) in adults who experience back pain in the past seven days compared to those without back pain during the same period.^16^ A study using a large health plan dataset from the United States compared the prevalence of chronic conditions between adults who have sought medical care for back pain and adults with no diagnosis of back pain.^50^ The prevalence of rheumatism (40.3 % versus 11.9 %), arthritis (34.4 % versus 10.9 %), osteoarthritis (14.2 % versus 3.8 %) and depression (13.0 % versus 6.1 %) among adults who sought medical care for their back pain were higher than those with no diagnosis of back pain in the same database.^50^ The explanation for the coexistence of chronic back pain with other chronic conditions is still uncertain. Some of these conditions may share similar lifestyle risk factors, such as smoking,^51^ obesity ^12^and depressive symptoms^52^ which may explain the coexistence of back pain with these chronic conditions. Further longitudinal studies are needed to investigate whether there is any cause-effect relationship.

The high prevalence of multimorbidity found in this study aligns with the findings of previous studies conducted in upper income countries.^25^^,^^28^^,^^53^ National population data from Australia shows that 74 % of people with back pain aged 45 and over were classified as having a comorbidity (i.e., reported also having one or more - up to 9 - chronic conditions).^54^ Other studies investigating a higher number of comorbidities found higher prevalence estimates. For instance, data from Germany investigating the lifetime prevalence of 31 comorbidities among people who reported low back pain in the past week found a comorbidity prevalence of 92 %.^16^ Similarly, data from the United States found a multimorbidity prevalence of 87 % (i.e., out of 20 possible comorbidities) among adults with chronic spinal pain in the past 12 months.^28^ Despite the use of different definitions for symptom duration and number and type of chronic conditions investigated, multimorbidity among adults with chronic back pain may be prevalent regardless of countries’ income level. One possible explanation for the high prevalence of multimorbidity is that chronic back pain may be part of a cluster of chronic conditions,^11^ which may share common risk factors, such as obesity^55^^,^^56^ and higher BMI.^57^

In the current study, the factors associated with comorbidity among people with chronic back pain were female sex, older age, higher BMI, and lower level of education. Our results aligned with evidence from previous studies.^21^^,^^58^, ^59^, ^60^, ^61^, ^62^, ^63^, ^64^, ^65^, ^66^ A recent systematic review with meta-analysis on the prevalence of multimorbidity in LMICs^61^ gives support for an association between multimorbidity, female sex and old age. These associations may be explained because females seek health services more often compared to men^67^ and most chronic conditions are more prevalent in older populations. Another finding was the association of multimorbidity with high BMI, because obesity is a known risk factor for back pain and a variety of chronic conditions, including diabetes,^55^ hypertension^56^ and cardiovascular disease.^56^ The mechanism might involve the inflammatory role of excess adipose tissue which has been documented,^68^^,^^69^ particularly in cardiovascular disease^56^^,^^57^ and cancer.^70^ Regarding the association between multimorbidity and lower education, similar results were found in a recent meta-analysis of cross-sectional studies.^58^ The authors showed that adults with lower levels of education had a greater chance of multimorbidity (OR=1.64, 95 %CI: 1.41, 1.91). The lack of access to health information and healthcare services among those with low levels of education may have a role in this association.^59^^,^^71^

The most frequent chronic health conditions among adults with chronic back pain observed in this study were cardiovascular disease, arthritis or rheumatism, and depression. A similar scenario regarding the type of comorbidity associated with back pain has been found in previous studies.^13^^,^^16^^,^^28^^,^^29^ Hypertension, osteoarthritis and/arthritis, mental and behavioural conditions (i.e., including depression) were respectively in first, second, and fifth place among the 10 most frequent conditions found in people with low back pain in Germany.^16^ Arthritis,^16^^,^^54^ circulatory system disease,^54^ mental and behavioural disorders,^29^ and hypertension^28^^,^^54^ were the four most prevalent conditions among adults with chronic back pain in previous studies carried out in Canada, Australia, and the United States. Among older adults with back pain, musculoskeletal conditions (such as pain and osteoarthritis),^27^ heart disease,^27^ and depression^72^ were among the 10 most prevalent conditions in recent cohort studies.^27^^,^^72^ Taken together, these findings suggest that cardiovascular diseases, musculoskeletal conditions, and mental health problems are the comorbidities that most frequently co-occur with chronic back pain.

We found that the high prevalence of multimorbidity is accompanied by activity limitations. While we found that a third of the sample (35.1 %) reported activity limitation ranging from “moderate” to “very severe”, the proportion of people in the multimorbidity group (40.6 %) was higher than in the no-comorbidities chronic back pain group (26.2 %). These results are in agreement with previous population-based studies showing that people with back pain reporting other comorbidities present with higher disability.^23^^,^^28^ In fact, the higher number of comorbidities is considered a poor prognostic factors for disability in acute^22^ and chronic low back pain.^26^^,^^72^ Most importantly, we identified specific comorbidities, such as arthritis or rheumatism, other lung diseases, depression, and cardiovascular diseases, more likely to be associated with activity limitation related to back problems. For instance, adults with chronic back pain and arthritis or rheumatism are twice as likely (OR=2.1; 95 % CI: 1.9, 2.4) to report higher levels of activity limitations compared to adults without this condition. Clinicians should be aware that most people with chronic back pain with these specific comorbidities may present with more disabling symptoms.

Although comorbidity is prevalent among people with chronic back pain, most clinical practice guidelines for back pain do not have clear recommendations on how patients with multimorbidity or patients with specific comorbidities should be managed in clinical practice.^73^ We now have more information about comorbidities that co-exist with chronic back pain that are associated with greater disability. Although the exact mechanism linking back pain with these range of comorbidities is unclear, we would argue that the presence of these conditions would need to be accounted for when designing or implementing treatment strategies. Future studies are still needed in this area to disentangle the best approaches for treating each of these combination comorbidities. While trials in this field are needed, preliminary evidence has started to emerge.^74^^,^^75^ Recent studies have tested different approaches, including healthy lifestyle interventions targeting a specific group of people with chronic back pain who are overweight or obese,^76^ interventions targeting the prevention of the comorbidity itself (i.e., major depression) in people with chronic back pain,^77^ or interventions testing different care pathways or multidisciplinary approaches.^78^

Our study has some limitations. Given the nature of our cross-sectional study design, we are unable to investigate causal relationships between chronic back pain and comorbidities. In addition, the definition of chronic back pain and number of comorbidities considered in the 2019 National Health Survey may limit direct comparisons across international studies. Finally, we collected self-reported data which may result in under- or overestimates of prevalence.^79^^,^^80^ Nevertheless, an important strength is that this population-based study uses recent representative sample data from an upper middle-income country.^39^

## Conclusions

Brazilian adults with chronic back pain show higher prevalence rates of non-communicable diseases compared to those without chronic back pain. We found that six in every ten adults with chronic back pain have one or more comorbidities. People with chronic back pain and comorbidity reported higher activity limitations related to back problems than those people who have only chronic back pain. Cardiovascular disease, arthritis or rheumatism, and depression were the most prevalent chronic health conditions in this population and were associated with higher levels of activity limitations related to chronic back pain. These findings suggest that the comorbidity patterns associated with chronic back pain are similar regardless of the countries’ income level. Further research should focus on designing and testing new interventions addressing or preventing the prevalent comorbidities that co-occur and make patients with chronic back pain more disabled.

## Declaration of competing interest

The authors declare that they have no conflict of interests.

## Appendix Supplementary materials

Image, application 1
