# Supplementary material for: Prevalence of non-communicable diseases, multimorbidity, and their impact on activity limitations among adults with chronic back pain: a national population-based study in a middle-income country
Source: Braz J Phys Ther. 2025 Aug 5;29(5):101241. doi: 10.1016/j.bjpt.2025.101241 (PMC12375196; doi:10.1016/j.bjpt.2025.101241)
Supplement: Supplementary file 1 [file mmc1.pdf]

Supplementary material. Description of the variables studied, data format in collection and coding of data in analysis

Participant characteristics	Collected data	Analyzed data (data code in analysis)
**Gender**	Woman or man	Dichotomous: female and male
**Age**	Age in years (continuous)	Categorical: 18- 29; 30-39; 40-49; 50-59; 60-69; 70-79; ≥ 80
**Skin color or ethnicity**[*]	White, Black, Asian, Brown, Indigenous	Categorical: white, black, yellow, brown, indigenous
**Educational levels**	School years	Categorical: Illiterate or incomplete primary school, complete primary school or incomplete high school, complete high school or incomplete college/university, complete college/university
**Per capita household income**		Categorical: up to 1 minimum wages, more than 1 to 3 minimum wages, more than 3 to 5 minimum wages, more than 5 minimum wages
**Household situation**	Urbano or rural	Dichotomous: urbano or rural
**Health Insurance (Do you have a private health plan)**	Do you have a private health plan?	Dichotomous: yes or no
**Smoking**	Non- smoking, ex-smoker, smoker	Categorical: non- smoking, ex-smoker, smoker
**Abusive alcohol consumption**	Abuse of alcoholic beverages 5 or more days a week	Dichotomous: yes or no
**BMI**	BMI calculated based on height (meters) and weight (kilograms)	m/kg^2 (continuous)<br><br>Categorical: Eutrophic (< 25); Overweight (≥25, < 30); Obesity (≥30)
**Presence of CBP**	Q84: "Do you have a chronic back problem, such as chronic back or neck pain, low back pain, sciatica, vertebrae or disc problems?"	Dichotomous: yes or no
**Estimated CBP symptom duration**	Symptom duration was calculated by subtracting the respondent's age when back pain started from the age at the time of the survey.	Dichotomous: greater than 1 year and equal or less than a year.
**Presence of cardiovascular disease**	Q2a: "Has a doctor ever diagnosed you with arterial hypertension (high blood pressure)?"	Dichotomous: yes or no

	Q63a: Has a doctor ever diagnosed you with a heart disease, such as a heart attack, angina, heart failure or other?"<br><br>Q68: Has a doctor ever diagnosed you with a stroke or stroke?"	
**Presence of diabetes**	Q30a: "Has a doctor ever diagnosed you with diabetes?"	Dichotomous: yes or no
**Presence of asthma**	Q74: "Has a doctor ever diagnosed you with asthma) or asthmatic bronchitis)?"	Dichotomous: yes or no
**Presence of arthritis or rheumatism**	Q79: "Has a doctor ever diagnosed you with arthritis or rheumatism?	Dichotomous: yes or no
**Presence of Depression**	Q92: "Has a doctor or mental health professional (such as a psychiatrist or psychologist) ever diagnosed you with depression?	Dichotomous: yes or no
**Presence of other mental health conditions**	Q110 a: "Has a doctor or mental health professional (such as a psychiatrist or psychologist) ever diagnosed you with another mental illness such as anxiety disorder, panic disorder, schizophrenia, bipolar disorder, psychosis or OCD (obsessive compulsive disorder) etc ?"	Dichotomous: yes or no
**Presence other lung disease**	Q116a: "Has a doctor ever diagnosed you with another chronic lung disease, such as pulmonary emphysema, chronic bronchitis or COPD (Chronic Obstructive Pulmonary Disease)?"	Dichotomous: yes or no
**Presence of cancer**	Q120: "Has a doctor ever diagnosed you with cancer?"	Dichotomous: yes or no
**Activity limitation due to CBP**	Q87: "In general, to what degree does your back problem limit your usual activities (such as working, doing household chores, etc.?)"	Categorical: (1)No limitation; (2); Mild (3) Moderate; (4) Severe; (5) Very severe

BMI, body mass index; CBP, chronic back pain.

*Pesquisa Nacional de Saúde 2019. Instituto Brasileiro de Geografia e Estatística IBGE, Brazil. Accessed 24 Apr 2024. https://www.ibge.gov.br/estatisticas/sociais/saude/9160-pesquisa-nacional-de-saude.html?=&t=microdados; 2024
